# How to work with intangible software in public health systems: some experiences from India

**DOI:** 10.1186/s12961-022-00848-9

**Published:** 2022-05-07

**Authors:** Sudha Ramani, Rakesh Parashar, Nobhojit Roy, Arpana Kullu, Rakhal Gaitonde, Ramya Ananthakrishnan, Sanjida Arora, Shantanu Mishra, Amita Pitre, Deepika Saluja, Anupama Srinivasan, Anju Uppal, Prabir Bose, Vijayshree Yellappa, Sanjeev Kumar

**Affiliations:** 1 Oxford Policy Management, New Delhi, India; 2 Evidence Action, New Delhi, India; 3 CARE India, New Delhi, India; 4 Achutha Menon Centre for Health Science Studies, Thiruvananthapuram, Kerala 695011 India; 5 Resource Group for Education and Advocacy for Community Health (REACH), Chennai, India; 6 Center for Enquiry into Health and Allied Themes, Santacruz East, Mumbai, 400055 India; 7 Gender Justice, Oxfam India, New Delhi, India; 8 Women in Global Health, New Delhi, India; 9 Vikalp Kriya, Panaji, Goa India; 10 NITI Aayog, New Delhi, India; 11 Health Systems Transformation Platform, New Delhi, India

**Keywords:** Health systems strengthening, Intangible, Leadership, Awards, Supervision, India, Low- and middle-income countries, Competence, Power, Trust

## Abstract

This commentary focuses on “intangible software”, defined as the range of ideas, norms, values and issues of power or trust that affect the performance of health systems. While the need to work with intangible software within health systems is increasingly being recognized, the practical *hows* of doing so have been given less attention. In this commentary, we, a team of researchers and implementers from India, have tried to deliberate on these hows through a practice lens. We engage with four questions of current relevance to intangible software in the field of health policy and systems research (HPSR): (1) Is it possible to rewire intangible software in health systems? (2) What approaches have been attempted in the Indian public health system to rewire intangibles? (3) Have such approaches been evaluated? (4) What practical lessons can we offer from our experience on rewiring intangibles? From our perspective, approaches to rewiring intangible software recognize that people in health systems are capable of visioning, thinking, adapting to and leading change. These approaches attempt to challenge the often-unchallenged power hierarchies in health systems by allowing people to engage deeply with widely accepted norms and routinized actions. In this commentary, we have reported on such approaches from India under six categories: approaches intended to enable visioning and leading; approaches targeted at engaging with evidence better; approaches intended to help health workers navigate contextual complexities; approaches intended to build the cultural competence; approaches that recognize and reward performance; and approaches targeted at enabling collaborative work and breaking power hierarchies. Our collective experiences suggest that intangible software interventions work best when they are codesigned with various stakeholders, are contextually adapted in an iterative manner and are implemented in conjunction with structural improvements. Also, such interventions require long-term investments. Based on our experiences, we highlight the need for the following: (1) fostering more dialogue on this category of interventions among all stakeholders for cross-learning; (2) evaluating and publishing evidence on such interventions in nonconventional ways, with a focus on participatory learning; and (3) building ecosystems that allow experiential learnings on such interventions to be shared.

The field of health policy and systems research (HPSR) emphasizes thinking of health systems as complex and adaptive entities that are shaped by human agency and action [1, 2]. Seen through this lens, the capacities within a health system can be examined in terms of its hardware (human resources, infrastructure, financial inputs) and tangible software (regulations, formal processes, technical capacities), as well as intangible software (values, norms, attitudes and relationships)—the three being dynamic parts of a whole [3, 4] (see Fig. 1). The HPSR lens not only acknowledges the myriad interdependencies among these three components, but also emphasizes their embeddedness in diverse social and political contexts [1, 2].

**Fig. 1:**
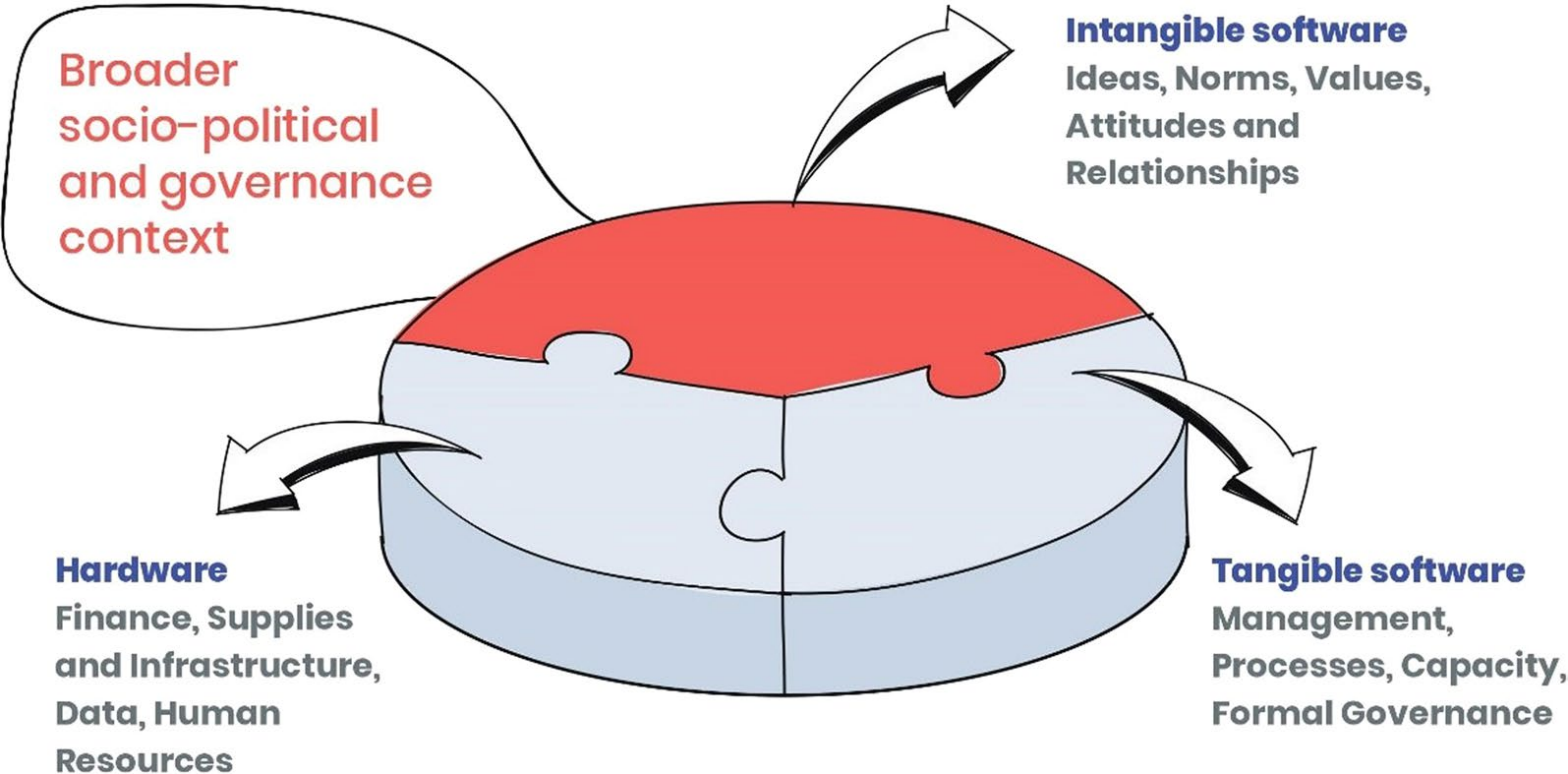
Conceptualizing health systems. Health systems conceptualized as comprising hardware and software, and situated in specific contexts [3, 4]

This commentary focuses on intangible software, defined as the range of ideas, norms, values and issues of power or trust that guide attitudes and behaviours in health systems, and that underpin the relationships between different health system actors [4]. The need to explicitly work with intangible elements in the health system has gained increased attention. Indeed, recent empirical studies in many different low- and middle-income countries (LMICs) have highlighted how perverse intangible software within health systems—including the demotivation of staff, lack of support and leadership, risk-averseness and hesitancy to implement new policies—undermines various system improvement efforts [5–9]. Further, evidence suggests when software elements are positively oriented—for example, in health facilities where trusting and collaborative relationships exist—the performance of health workers, as well as the quality of care provided by them, tends to be better [10].

Despite an increasing recognition of the need to work with intangible software in health systems, the more practical *hows* of doing so have been less clear. Some have argued that system reform efforts must begin with the intangibles, since changes in health systems and policies are, at the very core, determined by underlying values and ideas that shape the behaviours of people [11]. Others have contended that health policies and programmes must acknowledge and work with intangibles within more widely scoped system-strengthening efforts [4, 12, 13]. In general, however, there has been limited discussion of what approaches can be practically taken to rewire intangibles in health systems.

When we started working on this commentary, we found that very few papers could give us practical suggestions on approaches to rewiring intangible elements in health systems. Thus, in an attempt to engage with intangibles through a practice lens, a team of Indian colleagues (implementers, researchers, evaluators, and those who wear mixed hats) have compiled the various approaches to working with rewiring elements that we have come across in the course of our work. Drawing on our joint experiential knowledge in the Indian public health setting, with support from pertinent literature, we have tried to engage with four guiding questions:

1. Is it possible to rewire intangible software in health systems?
2. What approaches have been attempted in the Indian public health system to rewire intangibles?
3. Have these approaches been evaluated and proven worthy of programmatic investment?
4. What are our practical learnings on rewiring?

These guiding questions emerged from practice rather than theory. Our engagement with these guiding questions in this commentary is intended as a starting point to deeper empirical and theoretical work. We have discussed our thoughts on each of these questions below.

**1. Is it possible to rewire intangible software in the health system?**

In practice settings in India, we have often observed that perverse intangibles within health systems are considered as either unmodifiable or as too difficult to change. Very few initiatives to rewire intangibles have been tried, and even fewer have been documented. Hence, we have tried to make a case below as to why we consider intangibles to be amenable to change.

From our perspective, approaches to rewiring intangible software recognize and celebrate the human element in health systems. We see these approaches as being derived from an “actor-centric” philosophy that recognizes that people working in health systems are not automatons who carry out tasks mechanically. Rather, they are individuals with agency, who are capable of self-mastery, learning, visioning, collaborating, and adapting to and leading change [14, 15]. The actions and decisions of people are underpinned by their lifeworlds or lived realities [16, 17], which can be understood and reoriented in favour of broader health system goals. From this standpoint, it is possible to work with intangibles in order to improve performance and practice, and to strengthen health systems overall. Also, since individuals in the health system are embedded within formal and informal power hierarchies within health systems, working with intangibles is almost always an exercise in challenging existing power relationships.

There are documented examples across LMICs that highlight that working with intangibles is possible. The learning sites from Kenya and South Africa, which were attempts to systematize processes of reflection and sense-making within health systems through research partnerships, have shown promise in nurturing positive change [18]. From Guatemala, one study documents systemic changes achieved through a humanized version of supportive supervision to community health workers [19]. From India, the Ekjut trial, based on participatory learning and action (PLA) techniques, has demonstrated how such techniques can enhance community relationships with health systems [20, 21]. In general, documentation on working with intangibles is limited across LMICs, but the above examples do suggest that interventions to rewire intangibles have potential.

**2. What approaches have been attempted in India to rewire intangibles?**

From the authors’ collective knowledge of the Indian landscape, we have tried to compile a list of interventions that have been tried in India to enable the rewiring of perverse software in the public health system. We have compiled the interventions into six inductively derived categories, described in Box 1.^1^

**Table:** **Box 1: Approaches intended to rewire intangible software in health systems**

***Approaches intended to enable visioning and leading*** These approaches focus on enabling health workers to see value in their routine work and recognize themselves as leaders, and in doing so work towards recognizing and rewriting some of the systemically embedded routine scripts and practices that hinder change.	
***Approaches targeted at engaging with evidence better*** These approaches work on the premise that health workers at all levels need to engage with data with more intensity than as “fillers” of forms, and that if they are given an opportunity to do so, they have the capacity to critically engage with evidence and think of locally relevant solutions.	
***Approaches intended to help health workers navigate contextual complexities*** This cluster of ideas recognize that the day-to-day contexts that health workers have to reckon with are complex and challenging, and that there is a need to support health workers to handle such challenges, rather than blaming them for nonachievement or for being risk-averse.	
***Approaches intended to build the cultural competence of health workers and to enhance community relationships*** These approaches focus on sensitizing health workers to the needs of the community, providing a better sociocultural—and a more humane—orientation to health providers.	
***Approaches that recognize and reward performance ***These approaches are intended to recognize existing “exemplars” in the system, and to honour and reward them for their personal values, humane orientation, community relationships, innovations and commitment to their work. These awards are not meant to be for the achievement of numerical targets in the conventional sense.	
***Approaches targeted at enabling collaborative work and breaking power/gender hierarchies*** This set of approaches is targeted at breaking gender and power hierarchies in the system and enabling collaborative work across cadres or different teams of people.	

In Table 1, we present Indian examples of the approaches described in Box 1. We have included interventions attempted at both the managerial level and on the frontlines of the public health system. Only a few of these have been formally documented. The kind of interventions we have compiled here aimed at “first-order” culture change [22]: that is, they were about enabling people in the health system to do similar activities that they had been doing all along but *with a slight twist or a difference.*

**3. Have interventions on rewiring intangibles been evaluated and proven worthy of programmatic investment?**

This question gets asked by many well-intentioned governments and donor agencies who are interested in investing in rewiring interventions. These entities have expressed justifiable worries about the lack of concrete proof that such interventions are worthy of investment. We have attempted to put together our thoughts on this issue below.

There is a slowly growing body of evidence from different LMICs that points towards the promise of rewiring intangible software. The learning site approach in Kenya and South Africa, which uses PLA methods and encourages reflective practices, has highlighted the potential to improve social and emotional skills among health staff and to stimulate learning processes, and overall, better relationships in the system [18, 26]. The Health Workers for Change approach, which uses a series of participatory workshops to sensitize health workers to gender issues, has shown positive changes after these workshops in some places, but not all [27, 28]. Some interventions like supportive supervision, appreciative enquiry in systems and PLA have also been tried and declared as promising [19, 20] (also refer to Table 1 example 8). However, conventional proof of concepts—that is, evidence through conventional experimental methods where one can attribute change in community-level outcomes to particular interventions—may not always exist for the interventions described in this note.

**Table 1:** Examples of interventions that have been attempted to rewire intangible software in India

The approach	Example	Some learnings reported by the implementers
Approaches intended to enable visioning and leading		
1. *No one wants to feel like their job is meaningless*: informal gatherings and discussions to understand overarching policy visions and values	When a community monitoring intervention was initiated by the Society for Community Health Awareness Research and Action (SOCHARA) in Tamil Nadu, frontline workers were worried that this “monitoring” process would be used to unfairly accuse them of faults that they believed to be systemic. Hence, the workers were unwilling to cooperate. However, rather than start with an attitude of confrontation, staff from SOCHARA spent a lot of time just informally talking to health workers about notions of accountability and helping them understand why community monitoring processes had value and meaning. The informal discussions helped the health workers to accept the intervention	High-level support from the state authorities and government orders are needed. Not all people were willing to collaborate and be a part of this process, despite the existence of a government order. It was found that in some geographical pockets, people were more willing, and that these pockets could be used to demonstrate to the others who were hesitant the usefulness and value of this community monitoring process
2. Leadership trainings and nonclinical capacity-building initiatives	The Institute of Public Health has conducted district-level training programmes to build “champions” and “leaders” in Karnataka. There were reports of initial resistance to the training as there was a belief among the health workers that they were being tested during these training sessions. Hence, prior to the training, an extended rapport-building phase was necessary. A detailed evaluation of this training programme has been published [23]	There were anecdotes regarding resistance from the public sector staff to being trained as some of them felt that they were being tested. It took some time for the staff to relax into the programme. The evaluation found that the responses from different geographical divisions varied
3. *It is one champion who can nurture others*: Exposure of staff to inspirational examples	An ex-medical officer from a primary health centre shared that in the state she hailed from (anonymized), new recruits were exposed to exemplars or positive deviants in the public health system. This was done as a part of their induction training and aimed to provide new recruits with good role models to look up to, and, in the long term, to potentially add to the tribe of positive deviants in the health system	People learn both good and not-so-good practices from champions; thus, the champions must be carefully chosen. Even champions can’t work without basic infrastructural support
Approaches targeted at engaging with evidence better		
4. Helping routine data to speak differently through eye-opening data workshops	A series of workshops was conducted by the National Health Systems Resource Center on recognizing and engaging with health inequities in the data that health workers routinely encountered. These workshops gave people an opportunity to relook at routine data through a different lens—what the staff had earlier perceived as boring, routine data was used to enable a process of reflection	In some of the district pockets, the officials had attempted to recognize inequities and reach the more vulnerable in their programme in practical ways
5. Reinforcement of achievements locally using local data	One researcher-cum-implementor used facility-level data in a low-income state in India to engage in discussions with primary care nurses. Nurses looked at synthesized data and tried to reflect on their local achievements. The self-recognition of positive achievements seemed to play an important role in boosting local morale	The lack of supporting infrastructure is a deterrent to even the most motivated of nurses
Approaches targeted at navigating complexities in the context		
6. Buddy systems	This has been tried in some public medical college hospitals in different states in India. Buddy systems attempt to pair young recruits with champions or exemplars, who serve as mentors and support new workers through complex decision-making	The buddy system example here focuses on doctors, but it was suggested that it would be useful to have buddies across cadres. This system would be more effective if exemplars/stalwarts in the health systems came forward themselves to be “buddies” to younger staff
7. *Putting people in a safe space outside of work to reflect:* informal reflective spaces	A district-level official from one of the southern states in India conducted a series of residential workshops with the heads of different implementation bodies across sectors in order to break the hesitancy of people as regards collaborating across sectors. These workshops provided space for reflection and bonding away from work. No targets or checklists were used or discussed	Such workshops should be long-term, have repeated sessions over time, and preferably be residential—so that space to reflect and bond together without the interference of routine work is enhanced
Approaches intended to build the cultural competence of health workers and to enhance community relationships		
8. *Common understandings:* people and the system need to understand each other	The Ekjut trial on PLA took place in Jharkhand and Orissa. In this intervention, regular and iterative meetings were facilitated by accredited social health activists (ASHAs) (link workers associated with the Indian public health system) with women’s groups over 31 months [21]	The intervention needs to be participatory, even at the expense of time issues. Change is a time-consuming process
9. *How to talk to the community trainings:* explicit soft skills and communication trainings	In 2018–19, the Center for Enquiry into Health and Allied Themes (CEHAT) led a training intervention on domestic violence for health workers in two tertiary care hospitals in Maharashtra [24]. By codesigning the intervention with stakeholders, incorporating mixed-cadre training sessions and including explicit “soft skill” communication skills as part of the training, this training worked towards tweaking the culture within health facilities to be more sensitive to domestic violence issues	Programme staff realized that conveying some of these concepts, such as “equity” and “gender responsiveness”, during training was not straightforward. It was perceived by staff that attitudinal changes were easier to bring about in younger staff
Approaches that recognize and reward performance		
10. Social awards and incentives	The Kayakalp award scheme is run by the central health ministry in India and recognizes and awards health facilities that demonstrate their commitment to cleanliness, hygiene and infection control practices	Social awards have to be used carefully—for wrongly chosen award schemes (or corrupt awarding practices) can be demotivating
Approaches targeted at enabling collaborative work and breaking power/gender hierarchies		
11. Building confidence: training on soft skills, public speaking and speaking in English	Basic Health Services in Udaipur offered nurses formal leadership positions at primary care clinics [25]. The organization noticed that nurses were culturally hesitant about taking up leadership positions. The nurses were trained using a hybrid technical and soft skills module to build their rigour and confidence. It was reported that public-speaking skills, and particularly learning to speak in English, helped to boost nurses’ confidence	Structural and software interventions were needed to help nurses take up leadership positions. Leadership workshops must be seen only as one important step in trying to break down power hierarchies. Building leadership skills takes time
12. Sensitization workshops within the health system	The Resource Group for Education and Advocacy for Community Health (REACH) in Tamil Nadu has been supporting the Revised National Tuberculosis Control Programme [now the National TB Elimination Programme (NTEP)], to adopt a gendered lens to TB. As part of these efforts, a gender-responsive training curriculum was developed and piloted with NTEP in October 2020. The training used participatory techniques (including power walks) to sensitize people to power and gender hierarchies	An evidence base was needed to make a stronger case for gender responsiveness before embarking on the workshops, and this was achieved through a TB and gender assessment, followed by the adoption of a gender framework by the national programme. Such trainings must try to balance concepts along with granular action, and help participants understand how they can apply their learning in their specific roles

In fact, many of the intervention examples from our work that we have listed in Table 1 do not have decontextualized proofs of concept. For one, proofs of causal relationships between such interventions and community-level outcomes are not easy to establish. Even if these interventions have been evaluated or examined, the end results of these evaluations need to be viewed with caution and not taken as indicating blanket “success” or “failure” of the intervention (refer to Table 1, examples 1 to 5). We feel that combined successes and failures in the same intervention need to be accepted, and impact evaluations may not be able to capture these nuances. This is because rewiring interventions have complex change mechanisms that work or do not work depending on several factors in the context. An illustration of this complexity has been presented in an evaluation by Prashanth et al. (2014) of an intervention to strengthen district-level managerial skills undertaken by the Institute of Public Health, Bangalore. Conducted by applying a realist lens, this evaluation highlights several contextual factors that played a role in determining the ultimate impact of the managerial intervention, including staff turnover and the existence of infrastructural support. The authors of this evaluation point out that a decontextualized proof of concept may simply not exist for the kind of intervention they had tried; and applying a “what worked, why and for whom” approach was probably a more practical way to assess the merits of their efforts [23]. Such arguments have been put forth by Sardan and colleagues [13] as well, from their experience in sub-Saharan Africa. Sardan and colleagues have particularly emphasized the danger of copying intervention approaches without taking into account the subtle contextual nuances that made these approaches a success in the first place [13]. Cleary and colleagues offer similar arguments for evaluating a leadership intervention in South Africa through an “action-learning” design, which provided multiple opportunities for adapting and tailoring the intervention [29]. Thus, rather than traditional evaluation techniques (like measuring impact), evaluations that gather rich learnings and help to iteratively produce more potent and practical ways to rewire intangible software might be more useful for implementers of such approaches.

Another factor that makes the evaluation of rewiring approaches difficult is the timing. Many rewiring approaches aim at long-term, slow change, but usually evaluations of interventions tend to be carried out simultaneously or immediately after the intervention. A recent review of learning and development programmes in Africa notes that the effects of these programmes may become clear only after several years, and may not be visible in immediate assessments [30]. We concur on this point that we might not be able to capture the true effect of interventions on intangibles within more immediate time frames. We also feel that the lack of funding and expertise within programmes to conduct long-term evaluations is also a deterrent. That is, many a time, evaluators have to be externally hired for the purpose, and this is particularly disproportionately expensive in LMICs when the interventions being tried are small-scale and dependent on tight budgets.

**4. What works in practice? Some lessons from our experiences**

In the section we highlight some practical tips on working with intangible software.

***Hardware and software go hand in hand ***It is important that intangible software interventions are implemented hand in hand with improvements in hardware and tangible software. We give two examples below that illustrate the need for combined hardware–software interventions. Authors AU and PB were involved in conducting a series of training programmes for frontline counsellors in the public health system on the reproductive rights of women. These trainings emphasized inculcating counselling skills using a rights-based approach (rather than coercing women to adopt family planning methods). However, it was found that after receiving the training, the trained counsellors went to work in a context that was highly target-oriented, and the counsellors felt they had no room to practically apply the rights-based orientation that they had obtained during their training. In addition, it was reported that the hospital facility heads used counsellors for work other than counselling, and the counsellors, who were contractual employees, felt uncomfortable protesting against their diluted counselling roles. All this highlights that the usefulness of rewiring software approaches can be diluted if other structural systemic changes do not accompany these interventions (refer to Table 1, examples 3, 5 and 11). We share another learning on the same lines from Basic Health Services, a nongovernmental organization (NGO) in the state of Rajasthan in India which runs primary care clinics led by nurses. Nurses from these areas did not think of themselves as “leaders” of independent clinical work [25]. To change these attitudes, this NGO offered nurses formal roles that conferred more power on them (structural change). The NGO also held iterative technical and confidence-building training sessions to enable the nurses to think of themselves as change-makers and leaders (software change). We note that this combination of structural and software elements in this intervention, entwined deliberately, had the potential to change the existing status quo for nurses.

***Codesigning interventions with stakeholders ***Intangible software interventions work with complex ideas, ideologies and concepts that are not easy to work with. Hence, rewiring interventions can fail in their purpose if they are not codesigned with relevant stakeholders (refer to Table 1, examples 1, 6, 7, 9 and 10 that highlight need for codesigning and mention field-level suggestions for improving specific intangible interventions). One of our authors (anonymized) spoke of how the local health department in their area tried to set up a system whereby patients could rank a doctor from public primary care facilities after their visit, and share this ranking through a feedback box in the facility. The purpose of this intervention was to identify and motivate good doctors in the public sector. However, it was found that doctors tried to rig the voting system in their favour—since the doctors viewed the voting system as a form of ranking, rather than as a feedback mechanism. Thus, the system was not able to truly identify the “good” doctors through this intervention. This experience taught the managerial staff that rewiring interventions need to be tweaked to the context, and one of the ways to do this is through the participation of local stakeholders right from the design stage of the intervention. Another example of this kind was noted by SA. SA, based on her experience of working on a codesigned curriculum for health workers on domestic violence, emphasized that codesigning interventions is not a one-off process. The NGO she worked with had conducted a domestic violence programme in 2018–19 that tried to sensitize health workers to the needs of women who face domestic violence [24]. Before this training was launched, the technical content had already been discussed with the health workers, and their inputs had been obtained. But during the training, a training facilitator used a fictitious example of a woman from an ethnic minority to illustrate the concept of vulnerability. This example was misconstrued by one participant, who took offence against being thought of as “vulnerable”. Following this incident, the content of the training was revised again to make it more sensitive to the participants’ feelings. SA emphasized that truly codesigning an intervention is an iterative process that is time-consuming and one that involves immense effort if it is to be done right.

***Each place might need a different “hook”, and not everything works everywhere*** Not everything works for everyone when it comes to modifying intangibles, and this limitation has to be accepted. This learning can be seen across almost all interventions in Table 1. If we believe that people are unique and are bound to use agency differently, we need to enable the use of this agency for positive change. But, at the same time, we need to accept the inherent *nonuniformity* that is bound to surface in our enabling efforts. For instance, one of our authors (anonymized) shared the experience of being involved in a national-level training workshop. Among the trainees, many did not incorporate new learnings in their practice, but others seriously attempted to change some existing managerial practices in accordance with the new learnings and demonstrated fantastic local-level results. The evaluation by Prashanth et al. (2014) also pointed to how each subdivision in a district responded differently to a management training programme, and it noted that the response of people in complex systems is not always predictable. Among us, we have noted the need to start with small changes and not be discouraged by uneven or nonuniform results. A tribe of “positive” change-makers needs to be built over time; it helps to start with a few who are more inclined and able to foster change, and eventually snowball from there.

***Nurturing spaces for reflection within existing routines*** If we think of health systems as complex adaptive systems, this implies that there are adaptive mechanisms within such systems that work to maintain the status quo—even when this status quo is widely acknowledged as deficient [31]. One way to help people question this status quo is to enable a process of reflection and thinking among health workers and managers. Reports of the learning sites’ experience have captured several mechanisms through which developing spaces for iterative reflection and learning within practice settings offer scope for building “everyday resilience” in health systems, by building three kinds of capacities—cognitive, behavioural and contextual [26, 32]. As a group, we believe that many people who join public services have good intentions and are intrinsically motivated to help patients; however, much of this enthusiasm gets chipped away due to tough work schedules and constrained support in work settings. Offering spaces for reflection can help health workers gain renewed vigour and hope, and can open up their minds to finding solutions (refer Table 1, examples 4, 6, 7 and 12 that highlight such attempts). These approaches can be facilitated by trusted external parties (researchers, NGOs, think tanks). We feel that approaches can also be piggybacked onto existing capacity-building/technical training sessions. For instance, some training sessions on soft skills (talking in English, public speaking, confidence-building discussions) can be added on to existing new-recruit induction trainings in primary health facilities or other routine monthly meetings. That is, these sessions need not be completely “new” activities, but rather routine ones with a slight twist in how they are conducted.

***It takes decades of patience, empathy and investments*** Intangible software interventions often deal with ideas and values that are deeply embedded in the social fabric, and changing these is not an easy task. Indeed, it is easier to change practices through incentives and protocols than to change underlying attitudes. Yet lasting change comes only with attitudinal change. The need for time and patience has been noted repeatedly (refer to Table 1, examples 2, 7, 8, 11 and 12 that reflect these points). RA and AS from REACH, in particular, have noted the need for empathy, along with patience, from their experience of working on gender and tuberculosis. They observed that within the national programme on tuberculosis, the managerial cadres were mostly male, and issues of “gender” were a very novel concept in these circles. Both RA and AS highlighted the need for patience and for empathy with people who are involved in the change process, and they noted that “change is never easy for anyone”. They emphasized the need for empathetic discussions, trust-building and bonding, along with hard evidence to bring about a “slow” change. All of us writing this paper have expressed similar sentiments, the general consensus being that the chances of achieving instant results through intangible software interventions are very low.

## Concluding thoughts

Complexity theories on systems thinking emphasize that bringing about change is a messy, nonlinear and unpredictable process and that change agents need to work with multiple underlying issues in health systems [12]. Despite recognition of complexity in the change process, we feel that in India, like in many other LMICs, most efforts to bring about change continue to focus on the tangible aspects of the health system. Our collective experiences show that intangible software interventions—that aim to change leadership behaviours, trust, motivation, power balance and the values of health system actors—are often considered to be risky ventures that may not yield predictable results. Difficulties in measuring the impact of such interventions, as well as the scarcity of publications in this area, seem to contribute further to the lack of confidence of funders and governments in these efforts. Not surprisingly, the current situation of health system programming in India does not appear to favour investments that seek to alter intangibles.

However, the examples of interventions from India discussed in this paper suggest that it is possible to attempt to rewire intangible software in health systems. Such interventions appear to work best when they are codesigned, contextually adapted and implemented in conjunction with structural or hardware improvements. It is important to keep in mind, however, that the road to rewiring intangibles, in local health systems or sub-systems, may often be long and iterative. As Kwamie and colleagues point out, we need “long-term, more reflective and potentially unpredictable approaches” to strengthening capacities in health systems [33]. Further, evidence on such interventions may need “complexity-sensitive” learning assessments that focus on experiential learnings, rather than objective evaluations. There is also potential to explore more embedded approaches to researching such interventions, wherein the ownership of evaluation and learning rests largely with decision-makers and implementers [34].

Since this commentary is intended as a “practice” paper, we have not focused on the theoretical underpinnings of the experiential lessons we have shared here. For instance, the learnings from our efforts can be linked to perspectives from cultural sociology, that highlight how cultural scripts and repertoires act as a toolkit to shape action, making change a difficult and nonlinear process [35]. Our findings can also be mapped to scholarship on organizations and institutions, that offers perspectives on how individual agency relates to formal and informal institutional structures. For instance, work from the field of new institutionalism [36], cybernetics such as the viable system model [37] and institutional logic perspectives [38] can enable further interrogation of the interventions that we have mentioned in this paper. We invite others to take our work further through deeper engagement with such theoretical perspectives.

### The way forward

We conclude this commentary with three issues that need attention with respect to rewiring intangible software in health systems.

One, we feel that the routine dialogue among governments, researchers, funders and implementers must encompass explicit discussions on intangible elements in health systems. We consider this important as these stakeholders routinely discuss resourcing (hardware) and formal processes (tangible software) for systems improvement, but side-line discussions on intangible software. This happens possibly because elements of intangible software are challenging to unpack, potentially sensitive and considered difficult to change. However, we believe that opening difficult dialogues on intangibles in formal decision-making spaces can help to develop a collective understanding of these ideas, as well as generate more funding and interest in this area.

Second, there is a need to build, evaluate and publish evidence on working with intangibles in diverse fora. Implementers often possess deep knowledge of intangibles and their workings in specific contexts. They make multiple structured as well as not-so-structured attempts to modify intangibles, as we observe from the experiences shared in this paper. This tacit knowledge is often unpublished and remains within specific implementer groups. We feel that systematic efforts to capture such experiential learnings on intangibles are needed.

Lastly, we feel the need for ecosystems—both nationally and across LMICs—in which experiential learnings on intangible software can be shared. Such ecosystems can be built around formal research–practice collaborations. Further, informal platforms such as communities of practice, online knowledge-sharing platforms and other such groups of actors can help to augment evidence generation and advocacy on intangible software.

## Data Availability

Not applicable.

## Annexure

We have appended an infographic that highlights various approaches to rewiring intangible software. This infographic is intended for better communication of study learnings to a wider audience.

## Abbreviations

CEHAT: Center for Enquiry into Health and Allied Themes
HPSR: Health policy and systems research
LMIC: Low- and middle-income countries
NGO: Nongovernmental organization
NTEP: National Tuberculosis Elimination Programme
PLA: Participatory learning and action
REACH: Resource Group for Education and Advocacy for Community Health
SOCHARA: Society for Community Health Awareness Research and Action

## Footnotes

1 In January 2020, a team at Oxford Policy Management (OPM) undertook a rapid exercise to identify a list of intangible software interventions that have been attempted in the public health system in India by connecting with several academicians, evaluators and implementors. We started with an initial list of people identified by the OPM team internally and snowballed from there (we contacted 47 organizations in total). We produced a series of 12 case studies on rewiring intangible interventions, based on our conversations with different people. In May 2020, the OPM team reconnected with interested stakeholders to coproduce this commentary. We jointly decided on four guiding questions and then reflected on these four questions using the case studies as the basis for inductively emerging themes. The team at OPM did the first round of the cross-case analysis and wrote the first draft of this commentary. Subsequently, all authors have commented and reviewed various sections of the paper verbally or through email. More details on the processes we followed can be obtained by writing to the corresponding author.
